# Lysosomal perturbations in human dopaminergic neurons derived from induced pluripotent stem cells with *PARK2* mutation

**DOI:** 10.1038/s41598-020-67091-6

**Published:** 2020-06-24

**Authors:** Justyna Okarmus, Helle Bogetofte, Sissel Ida Schmidt, Matias Ryding, Silvia García-López, Brent James Ryan, Alberto Martínez-Serrano, Poul Hyttel, Morten Meyer

**Affiliations:** 1 Department of Neurobiology Research, Institute of Molecular Medicine, University of Southern Denmark, J.B. Winsløws Vej 21, 5000 Odense C, Denmark; 2 Department of Molecular Biology and Center of Molecular Biology Severo Ochoa, Autonomous University of Madrid-C.S.I.C Campus Cantoblanco, Madrid, Spain; 3 Oxford Parkinson’s Disease Centre, Department of Physiology, Anatomy and Genetics, University of Oxford, Oxford, United Kingdom; 4 Department of Veterinary and Animal Sciences, Faculty of Health and Medical Sciences, University of Copenhagen, Grønnegaardsvej 7, 1870 Frederiksberg C, Denmark; 5 BRIDGE – Brain Research Inter-Disciplinary Guided Excellence, Department of Clinical Research, University of Southern Denmark, J.B. Winsløws Vej 19, 5000 Odense C, Denmark

**Keywords:** Parkinson's disease, Mechanisms of disease, Mechanisms of disease, Mechanisms of disease, Parkinson's disease

## Abstract

Mutations in the *PARK2* gene encoding parkin, an E3 ubiquitin ligase, are associated with autosomal recessive early-onset Parkinson’s disease (PD). While parkin has been implicated in the regulation of mitophagy and proteasomal degradation, the precise mechanism leading to neurodegeneration in both sporadic and familial PD upon parkin loss-of-function remains unknown. Cultures of isogenic induced pluripotent stem cell (iPSC) lines with and without *PARK2* knockout (KO) enable mechanistic studies of the effect of parkin deficiency in human dopaminergic neurons. We used such cells to investigate the impact of *PARK2* KO on the lysosomal compartment and found a clear link between parkin deficiency and lysosomal alterations. *PARK2* KO neurons exhibited a perturbed lysosomal morphology with enlarged electron-lucent lysosomes and an increased lysosomal content, which was exacerbated by mitochondrial stress and could be ameliorated by antioxidant treatment. We also found decreased lysosomal enzyme activity and autophagic perturbations, suggesting an impairment of the autophagy-lysosomal pathway in parkin-deficient cells. Interestingly, activity of the GBA-encoded enzyme, β-glucocerebrosidase, was increased, suggesting the existence of a compensatory mechanism. In conclusion, our data provide a unique characterization of the morphology, content, and function of lysosomes in *PARK2* KO neurons and reveal an important new connection between mitochondrial dysfunction and lysosomal dysregulation in PD pathogenesis.

## Introduction

Parkinson’s disease (PD) is a progressive neurodegenerative disorder affecting 1–2% of the population. Although most PD patients develop late-onset sporadic disease, a subpopulation of patients develops early-onset or familial PD forms associated with various genetic mutations. Studying the effects of these mutations can provide valuable insights into the molecular pathways and mechanisms that lead to degeneration of dopaminergic neurons in PD^1–4^.

Mutations in the *PARK2* gene, encoding the protein parkin, have been identified as the most common cause of autosomal recessive early-onset PD and have underlined the importance of mitochondrial dysfunction in PD pathogenesis^5–7^. Parkin is a multifunctional E3 ubiquitin ligase involved in several cellular processes. Parkin-mediated ubiquitination of mitochondrial proteins^8–11^ triggers clearance of impaired mitochondria through the autophagy-lysosome pathway (ALP)^12^. Lysosomes are organelles specialized for degrading macromolecules derived from the extracellular space through endocytosis or phagocytosis, or from the cytoplasm through autophagy. In recent years, the pathological importance of lysosomes has been indicated by a rapidly growing number of human disorders linked to defects in lysosomal function including PD^13,14^ in which non-degraded lipids and misfolded proteins accumulate. Mutations in the GBA gene, coding for the lysosomal glycohydrolase β-glucocerebrosidase (GCase), cause Gaucher’s disease and several studies have reported GBA mutations as the numerically greatest genetic risk factor for PD^15–17^.

A number of studies point to an interplay between mitochondrial homeostasis and proper lysosomal function. Diseases caused by mutations of ALP proteins often exhibit mitochondrial defects as well^18–20^. Of relevance for PD, loss of GCase activity leads to mitochondrial dysfunction indicating that impaired lysosomal function negatively impacts mitochondria^15^. Supporting this, autophagy-enhancing drugs such as rapamycin have neuroprotective effects against the mitochondrial complex I inhibitor rotenone in cellular models of PD^21^. Interestingly, mitochondrial dysfunction induced by rotenone treatment alters the expression of lysosomal genes, perhaps because mitophagy induction regulates mitochondrial and lysosomal biogenesis through nuclear translocation of transcription factors^22,23^. Recent studies have documented mitochondria-lysosome membrane contact sites, which enable bidirectional regulation of mitochondrial and lysosomal dynamics, and have demonstrated how mitochondrial impairment supresses autophagic flux, suggesting a complex mutual relationship between these two cellular compartments^24–28^.

The exact relationship between mitochondrial and lysosomal function in PD is not well defined^23,25,26^, however, and its role in the pathogenic process remains uncertain. By studying the lysosomal compartment and function in the context of parkin deficiency, we sought to address whether chronic mitochondrial dysfunction causes lysosomal impairment, contributing to PD pathogenesis. For this purpose, we studied isogenic iPSC-derived neuronal cultures with and without *PARK2* mutation, which as we have recently shown, leads to several mitochondrial defects^29^. Parkin deficiency resulted in a number of perturbations including altered lysosomal content, morphology, and function as well as autophagic changes. This indicates a link between parkin deficiency and lysosomal disturbances.

## Results

### Identical differentiation potentials of *PARK2* KO iPSCs and control lines

To study the disease mechanism underlining *PARK2*-mediated PD, we analysed two isogenic iPSC lines that were created from a healthy control iPSC line, where KO of the *PARK2* gene was created by zinc finger nuclease gene editing technology^30^. Detailed cell line information is reported in our recent studies^29,31^.

**Figure 1:**
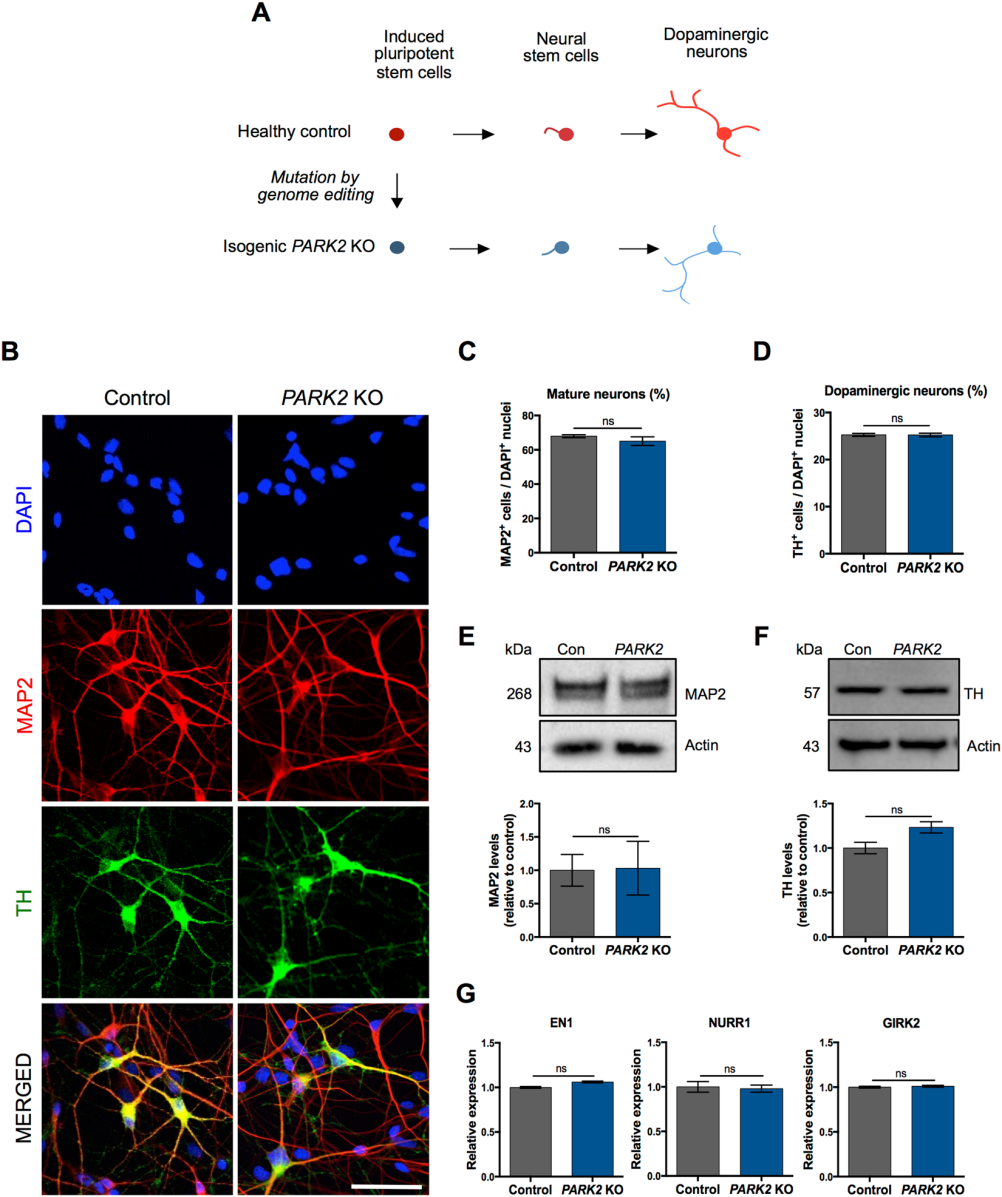
General characterization of neurons derived from *PARK2* KO and healthy isogenic induced pluripotent stem cells (iPSCs). (**A**) Graphical overview of the differentiation of iPSCs to neural stem cells (NSCs) and fully committed dopaminergic neurons. (**B**) Immunofluorescence analysis of MAP2 (mature neurons, red) and TH (dopaminergic neurons, green) expression in *PARK2* KO iPSC and control iPSC lines at day 25 of differentiation. Cell nuclei are visualized using DAPI (blue). Representative pictures are shown. Scale bar: 50 µm. (**C**,**D**) Quantitative assessment of (**C**) MAP2 + mature neurons and **(D)** TH + dopaminergic neurons in *PARK2* KO and control iPSC lines. Data are presented as mean ± SEM, n = 15, 5 independent differentiations. (**E**,**F**) Western blotting and densitometry analysis of (**E**) MAP2 and (**F**) TH protein expression levels in *PARK2* KO and control iPSC lines. Protein expression levels were normalized to α-actin. Full-length blots are presented in Supplementary Fig. S1. Data are presented as mean ± SEM, n = 3, 3 independent experiments. (**G**) qRT-PCR analysis for midbrain/dopaminergic markers (EN1, NURR1, and GIRK2). GAPDH, 18S, HPRT were used as endogenous references. Data were normalized to control levels and presented as mean ± SEM, n = 4, 2 independent experiments.

*PARK2* KO and isogenic control iPSC-derived neuronal stem cells (NSCs) were differentiated simultaneously to assess the efficiency of midbrain dopaminergic neuron yield (Fig. 1A). Figure 1B shows representative immunofluorescence pictures of cultures differentiated for 25 days, revealing a large percentage of MAP2 + mature neurons with distinct cell bodies and long branched processes forming highly interconnected networks. No apparent difference in the percentage of mature neurons was observed (control: 69.8 ± 1.0%, *PARK2*: 68.4 ± 0.9%) (Fig. 1C). The differentiated cells were also positive for NeuN and synaptophysin markers, which confirmed their maturity^29,31^. Many of the generated neurons co-localized with the dopaminergic marker tyrosine hydroxylase (TH), which is the rate-limiting enzyme in the production of dopamine. No apparent difference in the amount of TH + dopaminergic neurons was observed between cell lines (control: 25.25 ± 1.0%, *PARK2*: 24.6 ± 1.1%) (Fig. 1D). We also found GABAergic+ neurons and a small population of GFAP + astrocytes in the cultures^31^. Western blot analysis showed similar amounts of MAP2 and TH protein expression between control and *PARK2* KO cell lines, confirming the immunofluorescence staining (Fig. 1E,F). qRT-PCR analysis detected the presence of midbrain dopaminergic specific markers (*EN1, NURR1, GIRK2*) in the differentiated neuronal cultures (Fig. 1G). Both lines showed comparable expression levels with no significant differences. These data indicate that *PARK2* KO does not affect the neuronal differentiation potential of the iPSC-derived NSCs, as both the *PARK2* KO and isogenic control lines were equally efficient in generating midbrain dopaminergic neurons.

### Proteomic changes and increased lysosomal content in *PARK2* KO neurons

**Figure 2:**
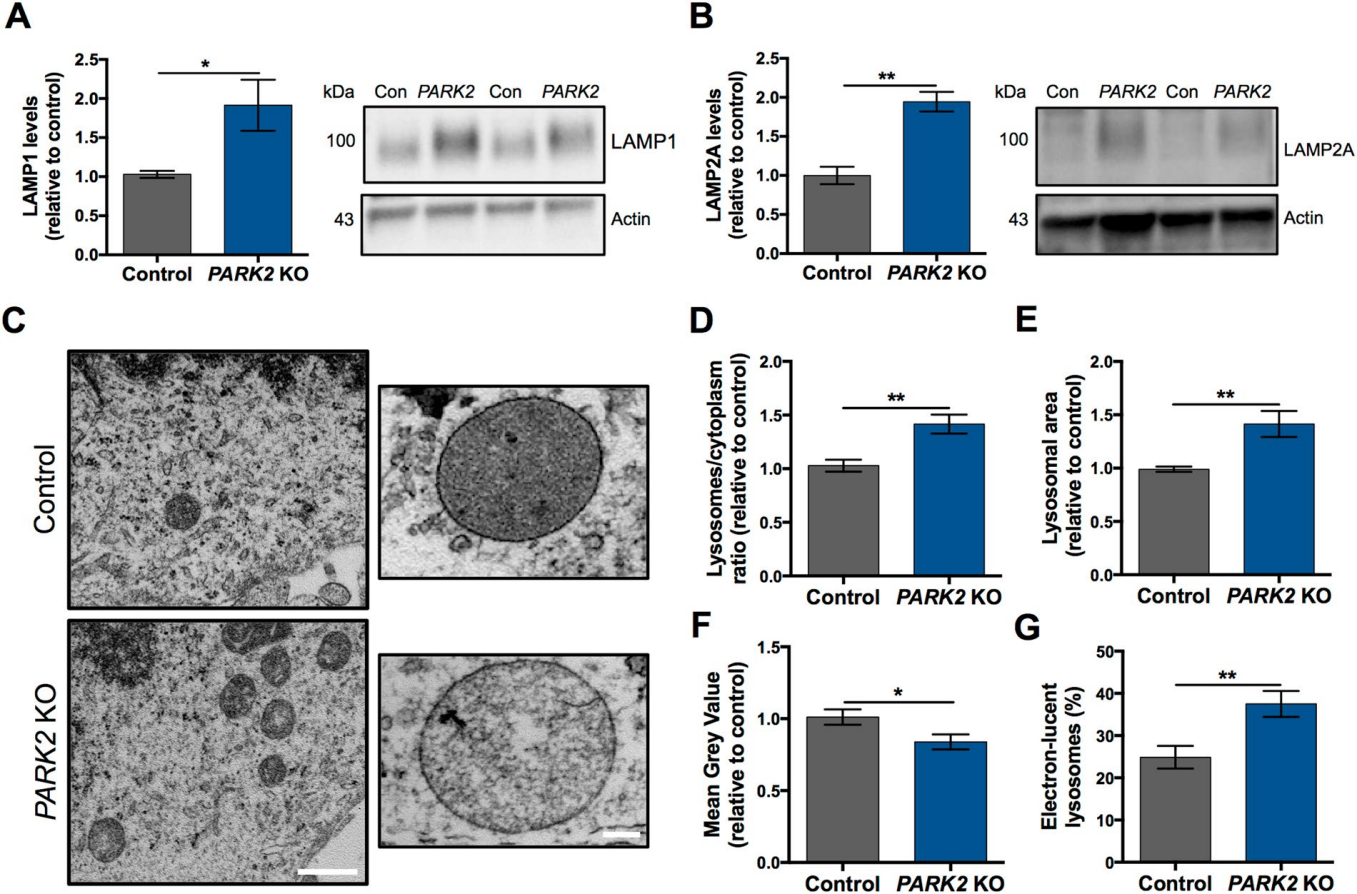
*PARK2* KO neurons exhibit aberrant lysosomes. (**A**,**B**) Western blotting analysis of the abundance of lysosomal markers, (**A**) LAMP1 and (**B**) LAMP2A. Expression levels were normalized to β-actin and are shown relative to control neurons. Full-length blots are presented in Supplementary Fig. S2. Data are presented as mean ± SEM of 3 independent differentiations (n = 3). Student’s t-test, *p < 0.05, and **p < 0.01. (**C**) Representative TEM micrographs showing the ultrastructure of lysosomes in healthy control (upper images) and *PARK2* KO (bottom images) neurons. Scale bars: 1 µm, 100 nm. (**D**) Quantification of the lysosomal abundance relative to cytoplasm showing lysosomal accumulation in *PARK2* KO neurons. (**E**) Lysosomal size, as a measurement of average area of the individual lysosomes, was increased in *PARK2* KO neurons. (**F**) Mean Grey Value of individual lysosomes was reduced in *PARK2* KO neurons. (**G**) Percentage of electron-lucent lysosomes among all lysosomes in a given neuronal population. Data are presented as mean ± SEM, n = 5–6 TEM grids, 3 independent differentiations (in total 45 TEM micrographs per cell line were analysed). Student’s t-test, *p < 0.05, and **p < 0.01.

As earlier reported, we subjected *PARK2* KO and isogenic control neuronal cultures to a mass spectrometry-based proteomic analysis that led to the identification and quantification of a large number of proteins (dataset accessible through the ProteomeXchange Consortium with the identifier PXD008894). We described dysregulation of 119 mitochondrial proteins in neurons with *PARK2* KO. The protein changes indicated disturbances in oxidative stress defense, mitochondrial respiration and morphology, which were confirmed by functional assays^29^. Based on this recently published dataset^29^, we also detected significant changes in the levels of 22 lysosomal proteins in *PARK2* KO neurons (Table 1). A number of these proteins were important for vesicle-mediated protein trafficking through the endosomal-lysosomal system and for the ALP pathway (Table 1). Lysosomal perturbations were thus present in the *PARK2* KO neurons and led us to examine the overall lysosomal content of *PARK2* KO neurons. Western blotting showed that levels of lysosomal-associated membrane protein 1 and 2A (LAMP1/2A), which are often used as general markers for lysosomes, were significantly elevated (Fig. 2A,B), pointing to an overall increased lysosomal content in the *PARK2* KO neurons.

**Table 1:** Changes in lysosomal proteins identified by proteomic analysis.

Accession	Gene name	Description	*PARK2* KO/Control	q-value	Unique Peptides
P27449	ATP6V0C	V-type proton ATPase 16 kDa proteolipid subunit	**1.56**	0.005	4
Q6IQ22	RAB12	Ras-related protein Rab-12	**1.31**	0.014	9
Q9Y5W7	SNX14	Sorting nexin-14	**1.30**	0.018	2
Q8IY95	TMEM192	Transmembrane protein 192	**1.25**	0.025	2
O15400	STX7	Syntaxin-7	**1.23**	0.036	13
Q96GC9	VMP1	Vacuole membrane protein 1	**1.21**	0.035	3
Q9H267	VPS33B	Vacuolar protein sorting-associated protein 33B	**0.87**	0.039	7
P78537	BLOC1S1	Biogenesis of lysosome-related organelles complex 1 subunit 1	**0.85**	0.042	3
Q969J3	BORCS5	BLOC-1 related complex subunit 5	**0.85**	0.037	2
Q9H305	CDIP1	Cell death-inducing p53-target protein 1	**0.84**	0.028	2
P07858	CTSB	Cathepsin B	**0.84**	0.047	2
Q9UN37	VPS4A	Vacuolar protein sorting-associated protein 4A	**0.83**	0.036	8
P86790	CCZ1B	Vacuolar fusion protein CCZ1 homolog B	**0.82**	0.020	3
Q8TAF3	WDR48	WD repeat-containing protein 48	**0.81**	0.042	8
O14964	HGS	Hepatocyte growth factor-regulated tyrosine kinase substrate	**0.79**	0.015	5
P61073	CXCR4	C-X-C chemokine receptor type 4	**0.79**	0.016	3
O43237	DYNC1LI2	Cytoplasmic dynein 1 light intermediate chain 2	**0.74**	0.017	19
Q01484	ANK2	Ankyrin-2	**0.72**	0.009	167
P61916	NPC2	NPC intracellular cholesterol transporter 2	**0.69**	0.020	3
P60520	GABARAPL2	Gamma-aminobutyric acid receptor-associated protein-like 2	**0.67**	0.004	6
P98164	LRP2	Low-density lipoprotein receptor-related protein 2	**0.63**	0.003	16
Q9H492	MAP1LC3A	Microtubule-associated proteins 1 A/1B light chain 3 A	**0.57**	0.003	2

Ratio of lysosomal protein levels in *PARK2* KO neurons compared to that of controls; q-value (FDR-adjusted p-value) and number of unique peptides, n = 3, data based on three independent experiments.

### TEM evidence for lysosomal abnormalities in *PARK2* KO neurons

To further investigate the lysosomal perturbations suggested by the proteomic analysis, we applied transmission electron microscopy (TEM) analysis, which revealed several ultrastructural disturbances within lysosome-like structures. Lysosomes are small electron-dense spherical organelles that are enclosed by a single membrane and have a diameter of about 0.5–1.0 µm^32^. Ultrastructural assessment of the *PARK2* KO and healthy control neurons detected clear differences in lysosomal morphology between the two cell lines. Lysosomes in healthy control neurons were uniform and displayed a homogeneous electron-dense content, whereas lysosomes in neurons with *PARK2* KO were more heterogeneous as a substantial proportion of them presented a less electron-dense content with large translucent areas (Fig. 2C). Quantitative analysis showed a significant increase in the number of lysosomes relative to the total cytoplasmic area in *PARK2* KO neurons compared to healthy isogenic controls (38% increase, p < 0.01) (Fig. 2D). The area of the individual lysosomes was increased for *PARK2* KO neurons by approximately 40% (p < 0.01) compared to controls (Fig. 2E), thus verifying the presence of enlarged lysosomes observed for *PARK2* KO neurons. In addition, the mean grey value (a measure of lysosomal electron-density) was significantly reduced for *PARK2* KO neurons (20% decrease, p < 0.05) (Fig. 2F). This led us to categorize lysosomes into two groups according to their TEM morphology, electron-lucent (light) and electron-dense (dark), and we found significantly more electron-lucent lysosomes in the *PARK2* KO neurons compared to controls (37.5% vs. 24.8%, respectively, p < 0.01) (Fig. 2G). Overall, TEM analysis revealed significant morphological changes in lysosomes between *PARK2* KO neurons and isogenic control neurons. Lysosomes appeared to be more abundant, larger, and more electron-lucent (lighter) in *PARK2* KO neurons than in controls.

### Altered lysosomal abundance in mature *PARK2* KO neurons

**Figure 3:**
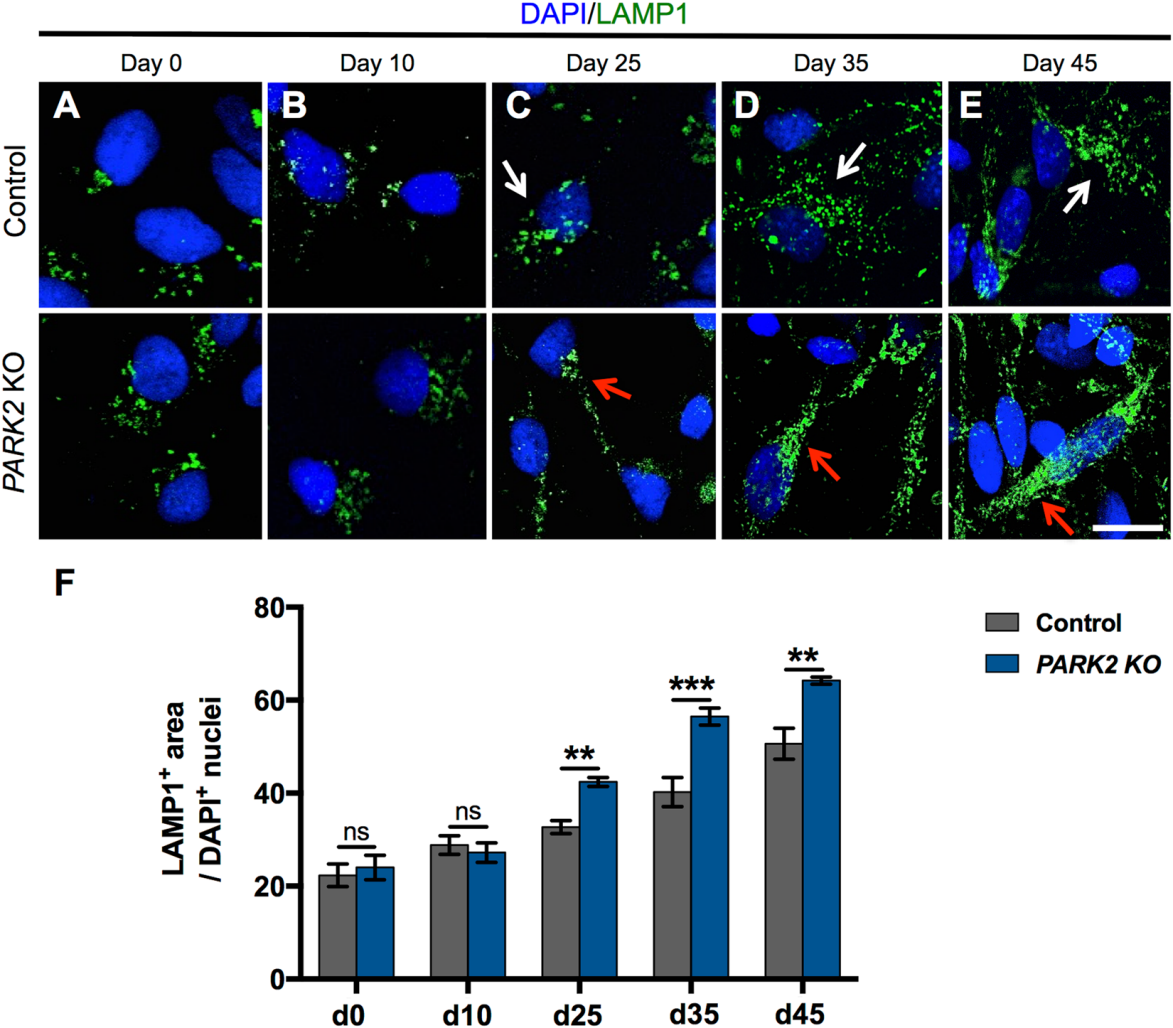
Increased lysosomal area in mature *PARK2* KO neurons. (**A**–**E**) Temporal changes in LAMP1 (green) immunoreactivity in iPSC-derived neurons from a healthy control and *PARK*2 KO cells. Cell nuclei were visualized using DAPI (blue). Scale bar: 20 µm. (**F**) Quantification of the area of LAMP1 + lysosomes normalized to the number of DAPI + nuclei showing no difference between *PARK2* KO and control cells at days 0 and 10, and a significant increase at day 25 and later time points. Data are presented as mean ± SEM of 3 independent differentiations (n = 9). Student’s t-test, **p < 0.01, ***p < 0.001, ns: not significant.

To investigate the time course of the lysosomal accumulation during the differentiation of *PARK2* KO neurons, we examined the abundance of lysosomes at different time points using immunofluorescence staining with the lysosomal marker LAMP1 (Fig. 3A–E). We observed no significant differences in the pattern of LAMP1 staining between *PARK2* KO and isogenic control neurons in the early stages of differentiation at day 0 (neural stem cell stage) and 10 (progenitor cell stage) (Fig. 3A,B,F). After 25 days of differentiation, when approximately 70% of the neurons were mature (Fig. 1C), *PARK2* KO neurons had a significantly increased area of lysosomes per cell, consistent with the obtained TEM data. Thus the area of LAMP1 + staining per DAPI + nuclei in *PARK2* KO neurons was approximately 25% larger than that in control neurons (Fig. 3C,F). The lysosomal area increased in both cell lines proportional to the time of differentiation, through with greater lysosomal accumulation in *PARK2* KO neurons (Fig. 3A–F). Moreover, at the later stages of differentiation, lysosomes were well resolved as puncta in healthy control neurons (Fig. 3C–E*, upper panel, white arrows*). In contrast, lysosomes appeared enlarged and clustered in *PARK2* KO cells (Fig. 3C–E*, lower panel, red arrows*). Altogether, these findings demonstrate that parkin dysfunction causes lysosomal accumulation and altered subcellular distribution in mature human *PARK2* KO neurons in a time-dependent manner.

### Mitochondrial uncoupling leads to lysosomal accumulation

**Figure 4:**
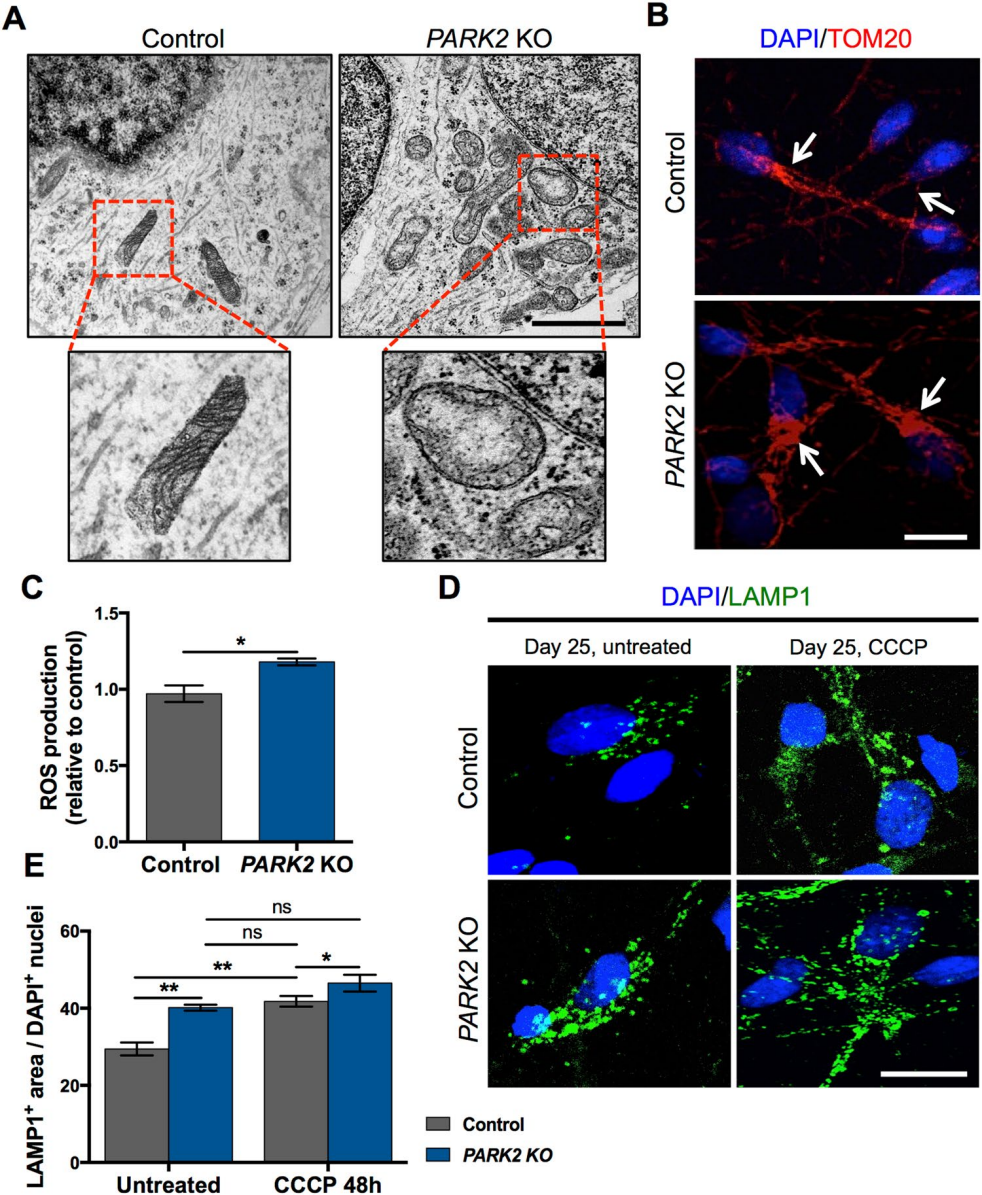
Mitochondrial and lysosomal alterations in *PARK2* KO neurons. (**A**) Longitudinally cut axon and mitochondria of control and *PARK2* KO neurons at day 25 of differentiation. Lower panels show enlarged pictures of the areas marked by red boxes in the upper panels. Scale bar: 1 µm. (**B**) TOM20 (red) and DAPI (blue) immunofluorescence staining confirming an increase in mitochondrial abundance, especially visible in the perinuclear area of *PARK2* KO neurons (marked with white arrows). Scale bar: 20 µm. (**C**) ROS production in control and *PARK2* KO neurons. Data presented as ± SEM, n = 12, 3 independent differentiations. Student’s t-test, *p < 0.05 (**D**) LAMP1 (green) and DAPI (blue) immunofluorescence staining of *PARK2* KO neurons and isogenic controls at day 25 after CCCP treatment (10 µM, 48 h). Scale bar: 20 µm. (**E**) Significant increase of the lysosomal area in both control and the *PARK2* KO neurons after CCCP exposure. Data presented as mean ± SEM, n = 9, 3 independent differentiations. Two-way ANOVA, *p < 0.05, **p < 0.01, ns: not significant.

The mitochondrial alterations in *PARK2* KO neurons, indicated by our proteomics study^29^, were confirmed by qualitative analysis of TEM pictures. Ultrastructural assessment of TEM micrographs revealed that mitochondria in *PARK2* KO neurons were often accumulated in the perinuclear region and they appeared swollen with a decreased matrix density and irregular cristae. In contrast, mitochondria in healthy, isogenic control neurons had a typical oval appearance with well-organized cristae and were distributed more homogeneously throughout the cell cytoplasm (Fig. 4A). In addition, a similar observation of increased mitochondrial content, especially visible in the perinuclear area of the mutated cells, was confirmed by immunofluorescence staining for the mitochondrial outer membrane protein TOM20 (Fig. 4B) as earlier reported^29^. Moreover, a significantly enhanced ROS production was detected in *PARK2* KO neurons (Fig. 4C).

To investigate whether the accumulation of lysosomes observed in *PARK2* KO neurons at day 25 was a direct consequence of mitochondrial impairment, we studied the lysosomal response to mitochondrial stress caused by *carbonyl cyanide m-chlorophenyl hydrazine* (CCCP), a proton ionophore that collapses the proton electrochemical gradient across membranes and is thus a potent uncoupler of respiratory chain function and oxidative phosphorylation^33^. To determine the optimal exposure time, the neurons were treated with 10 µM CCCP for 24, 48, 72 and 96 hrs, respectively. The number of cells did not change significantly over time upon CCCP treatment, however, there was a clear trend towards a decreased number of cells after 72 and 96 hrs of exposure (Supp. Fig. S5). Based on these results, we chose 48 h of exposure for the following experiments. This lead to an increase of the lysosomal area in control cells to a level similar to that of untreated *PARK2* KO neurons (41.8 vs. 40.15 arbitrary units, respectively, p = 0.98), strongly indicating that mitochondrial function impairment leads to lysosomal accumulation. CCCP treatment of *PARK2* KO neurons resulted in further enhancement of LAMP1 staining (Fig. 4D,E), consistent with the hypothesized mitochondrial dysfunction present in these cells and indicating that this is a general consequence of mitochondrial dysfunction. Taken together, these data show that exposure to mitochondrial stressors, like CCCP, results in elevated lysosomal area in both *PARK2* KO and healthy neurons. This provides further evidence of a causative link between mitochondrial dysfunction and lysosomal alterations.

### Perturbed lysosomal function and autophagic changes in *PARK2* KO neurons

**Figure 5:**
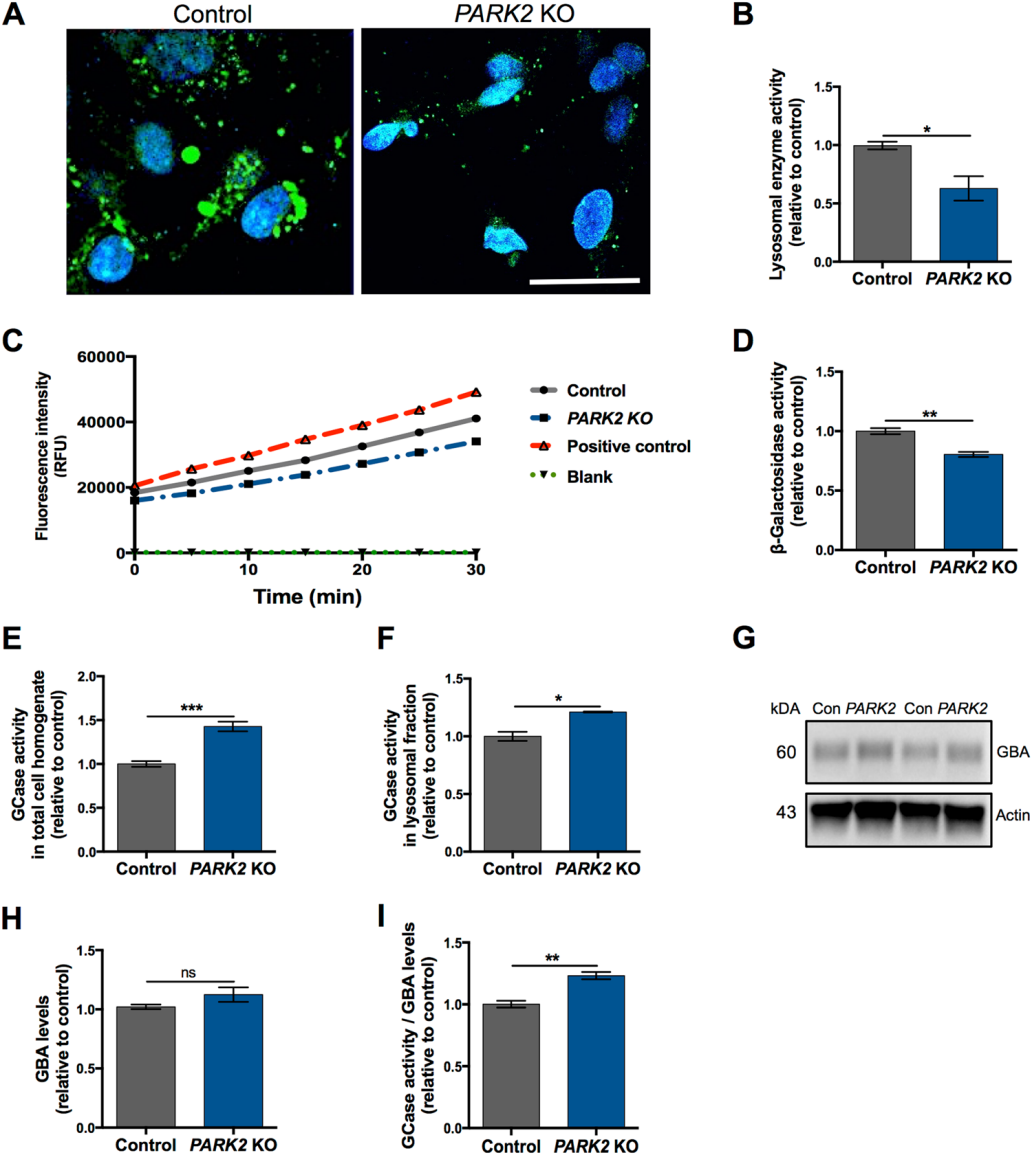
Perturbed lysosomal function in *PARK2* KO neurons. (**A**) General intracellular lysosomal enzyme activity manifested by generation of the fluorescence signal in *PARK2* KO and control neurons. Scale bar: 50 µm. (**B**) Quantification of the signal intensity showed a significant decrease in overall functional activity of lysosomal enzymes in *PARK2* KO neurons compared to controls. (**C**) Kinetics of enzymatic activity of β-galactosidase (β-Gal) based on relative fluorescence intensity versus time. Graph represents changes in the *PARK2* KO neurons (blue dashed line) and control neurons (grey solid line), positive control (red dashed line), and blank (green dot-dash line). (**D**) The specific β-Gal activity was significantly reduced for the *PARK2* KO neurons. Data presented as ± SEM, n = 6, 2 independent differentiations. Student’s t-test, *p < 0.05, **p < 0.01. (**E**,**F**) Glucocerebrosidase (GCase) enzyme activity was significantly increased for *PARK2* KO neurons compared to controls in both (**E**) total cell homogenate and (**F**) lysosomal fraction. (**G**,**H**) Western blotting showed no changes in GBA levels. Expression levels were normalized to β-actin and are shown relative to control neurons. Full-length blots are presented in Supplementary Fig. S3. (**I**) GCase enzyme activity normalized to GBA levels was significantly increased in *PARK2* KO neurons. Data presented as ± SEM, n = 4–6, 2–3 independent differentiations. Student’s t-test, *p < 0.05, **p < 0.01, ***p < 0.001, ns: not significant.

To determine whether the alterations in lysosome protein levels and overall organelle structure may impact lysosomal function, we performed several functional assays. We first assessed lysosomal intracellular activity (general enzyme activity) and found a significant >30% reduction (p < 0.05) in overall functional activity of lysosomes in *PARK2* KO neurons compared to controls (Fig. 5A,B). To further investigate the impairment of lysosomal function, we additionally monitored the activity of two essential lysosomal enzymes, β-galactosidase (β-Gal, Fig. 5C,D) and β-glucocerebrosidase (GCase, Fig. 5E–I). β-Gal activity was linear over the assay time, and a clear reduction was observed for the *PARK2* KO neurons compared to controls, whose pattern was more similar to the positive control included in the assay (Fig. 5C). Calculation of the specific β-Gal activity confirmed a reduced activity in the *PARK2* KO neurons compared to healthy isogenic controls (approximately 20% decrease, p < 0.01) (Fig. 5D). Contrarily, the activity of the lysosomal enzyme GCase, encoded by the PD-related *GBA* gene, was significantly increased in the *PARK2* KO neurons (approximately 42% increase in total cell homogenate, p < 0.001 and 21% increase in lysosomal fraction, p < 0.05) (Fig. 5E,F). As the protein levels of GCase were not significantly increased (Fig. 5G,H), the enhanced GCase activity was not simply an effect of increased lysosomal numbers or enlargement, but may reflect a compensatory functional activation (Fig. 5E–I).

**Figure 6:**
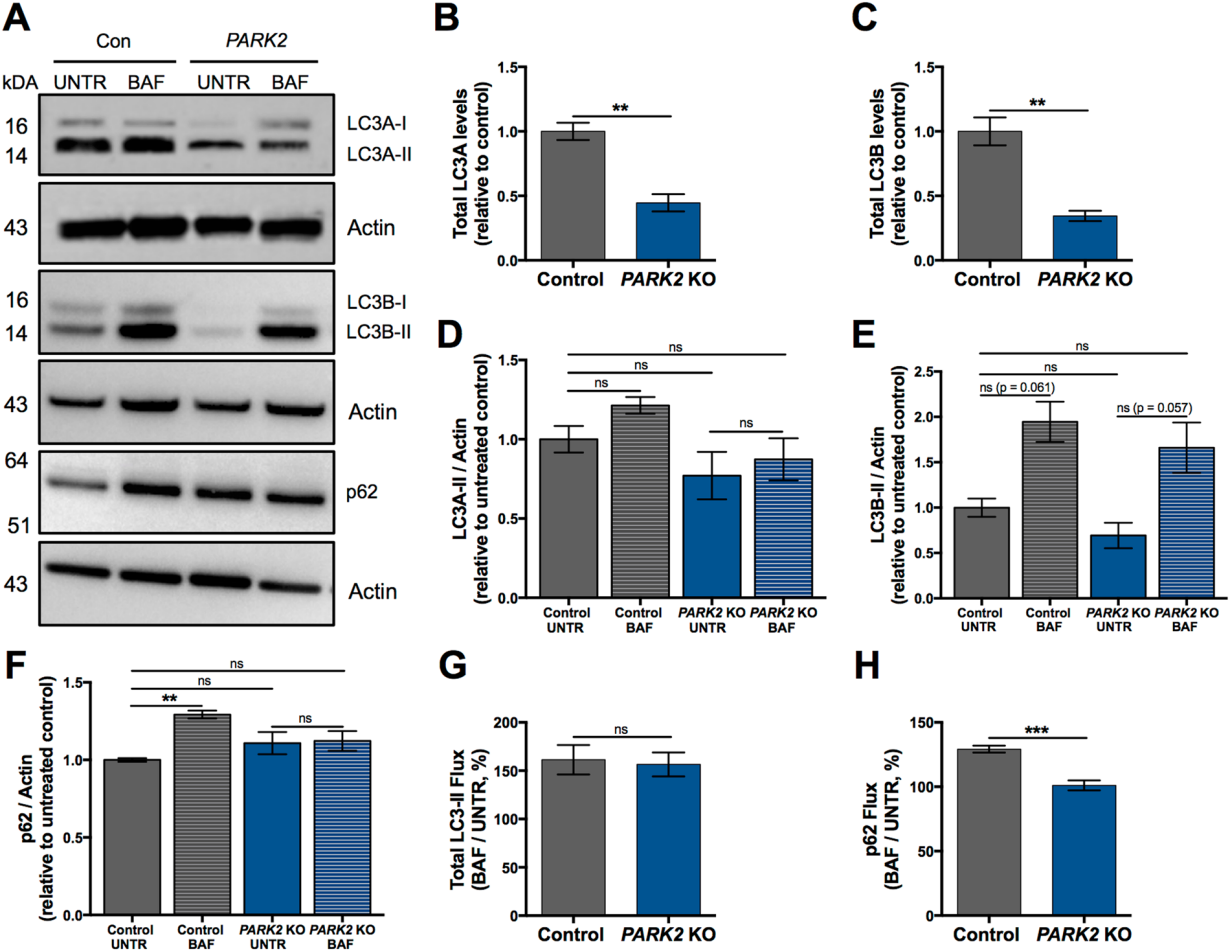
Autophagic changes in *PARK2* KO neurons. (**A**–**H**) Investigation of autophagy-related protein expression in *PARK2* KO neurons. (**A**) Differentiated cells were incubated with 10 nM bafilomycin A1 (BAF) for 6 h or with equivalent amount of DMSO as vehicle (UNTR), and Western blotting was performed for LC3A, LC3B and p62. Total (**B**) LC3A (LC3A-I + LC3A-II) and (**C**) LC3B (LC3B-I + LC3B-II) levels were normalized to actin. (**D**) LC3A-II, (**E**) LC3B-II and (**F**) p62 levels for each group were calculated by densitometric analysis and normalized to actin. Full-length blots are presented in Supplementary Fig. S4. (**G**) Total LC3-II flux and (**H**) p62 flux calculated as the ratios LC3-II (BAF)/LC3-II (UNTR) and p62 (BAF)/p62 (UNTR), respectively. Data are presented as mean ± SEM, n = 3–4, two independent differentiations. Student’s t-test (**B**,**C**,**G**,**H**) or one-way ANOVA (D-F), **p < 0.01, ***<0.001, ns: not significant.

In order to establish if the alterations detected in the lysosomal function also affected the autophagic process, as alluded to by the proteomic analysis (Table 1), we used Western blotting to analyse the expression of microtubule-associated proteins 1A/1B light chain 3A (LC3A, gene MAP1LC3A in Table 1), LC3B and p62 since these proteins are commonly used for the detection of autophagic activity^34–36^. The total amount of LC3A and LC3B proteins was significantly decreased in *PARK2* KO neurons at baseline, confirming the results of the proteomic analysis (Table 1, Fig. 6A–C). LC3-II (LC3A-II and LC3B-II) and p62 protein turnover was measured in the presence and absence of bafilomycin A1, which inhibits lysosomal acidification and fusion of autophagosomes with the lysosome. The levels of LC3A-II and LC3B-II were not significantly changed at baseline in *PARK2* KO neurons compared to controls. For both cell lines bafilomycin A1 treatment did not affect LC3A-II levels, while a trend towards increasing LC3B-II levels after treatment was observed, though this did not reach statistical significance (p = 0.061 for untreated vs. treated control neurons; p = 0.057 for untreated vs. treated *PARK2* KO neurons) (Fig. 6A,D,E). No significant change was detected between untreated control and *PARK2* KO neurons when analysing p62 expression. Control neurons showed a significant increase in the p62 level after bafilomycin A1 treatment, whereas this was not seen for *PARK2* KO neurons, where the protein level was unaltered after the treatment (Fig. 6A,F). Overall, the *PARK2* KO neurons showed unaffected total LC3-II flux and decreased p62 flux compared to healthy control neurons (Fig. 6G,H). Taken together, our data provide evidence of impaired lysosomal activity and autophagic disturbances in *PARK2* KO neurons.

### Antioxidant treatment ameliorates lysosomal accumulation in *PARK2* KO neurons

**Figure 7:**
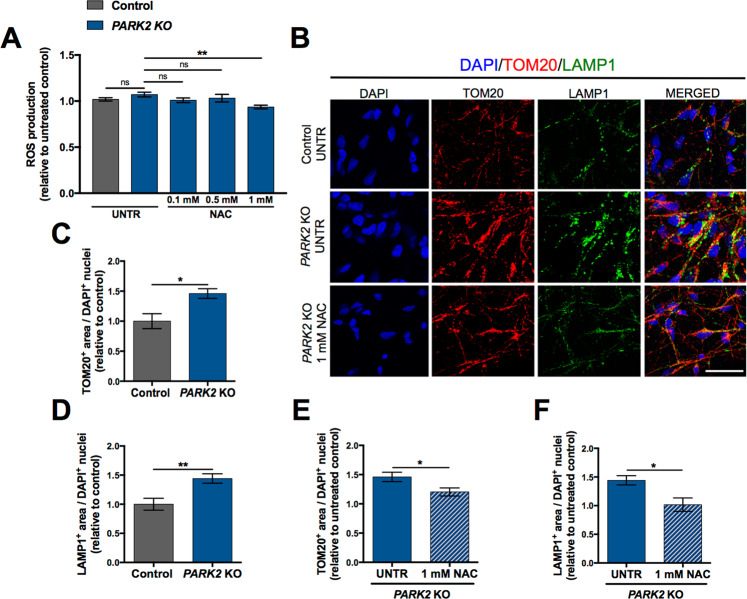
Effect of antioxidant treatment on *PARK2* KO neurons. (**A**) ROS production in *PARK2* KO neurons at day 26 after NAC treatment (0.1 mM, 0.5 mM and 1 mM) for 48 h. Data presented as ± SEM, n = 8, two independent differentiations. One-way ANOVA, **p < 0.01, ns: not significant. UNTR: untreated, NAC: N-Acetyl-L-Cysteine. (**B**) Representative immunofluorescence images of TOM20 (mitochondria, red) and LAMP1 (lysosomes, green) in untreated *PARK2* KO neurons and after NAC exposure for 48 h, compared to untreated healthy control cells. Cell nuclei are visualized using DAPI (blue). Scale bar: 50 µm. (**C**,**D**) Quantification of the area of (**C**) TOM20 + mitochondria and (**D**) LAMP1 + lysosomes normalized to the number of DAPI + nuclei in untreated *PARK2* KO neurons compared to healthy controls at day 26. (**E**,**F**) Quantitative assessment of (**E**) TOM20 + mitochondrial area and (**F**) LAMP1 + lysosomal area in *PARK2* KO cells after NAC (1 mM) treatment compared to untreated *PARK2* KO. Data are presented as mean ± SEM, n = 6, two independent differentiations. Student’s t-test, *p < 0.05, **p < 0.01 ns: not significant. UNTR: untreated, NAC: N-Acetyl-L-Cysteine.

To further investigate the connection between mitochondrial dysfunction and lysosomal disturbances, we tested whether antioxidant treatment in the form of *N-Acetyl-L-Cysteine* (NAC) by inhibiting ROS production could affect the lysosomal accumulation. Treatment duration time and applied doses were chosen based on the current literature^37^: neurons were treated with NAC (0.1 mM, 0.5 mM, 1 mM) for 48 h and subsequently ROS levels were determined. Surprisingly, we observed only a non-significant increase in ROS levels in *PARK2* KO neurons with this particular batch of cells. However, a significant reduction occurred after treatment with 1 mM NAC (Fig. 7A), suggesting an ability of this compound to mitigate oxidative stress. Next, we aimed to investigate whether the applied antioxidant treatment could restore mitochondrial and lysosomal levels in parkin-deficient cells. For this purpose, antioxidant-treated neurons were evaluated by double immunofluorescence staining for TOM20 and LAMP1. Full dose-response curves are included in the Supplementary Information (Supp. Fig. S6). We were able to confirm our earlier findings of increased levels of TOM20+ and LAMP1+ staining area in *PARK2* KO neurons when compared to controls (Fig. 7B–D). Interestingly, in *PARK2* KO neurons a significant decrease in mitochondrial and lysosomal areas was observed after exposure to 1 mM NAC when compared to untreated neurons (Fig. 7B,E,F). These data provide initial evidence of the possible beneficial effects of antioxidant treatment on both mitochondria and lysosomes, however, further research is required for accurate understanding.

## Discussion

The importance of parkin dysfunction in familial and sporadic PD is well established, but the exact mechanism and pathways are not well understood^6,38^. Parkin was first associated with mitochondrial function through studies in *Drosophila M*.^39^, but today we know that the parkin protein has multiple roles and that is crucial for the proper function of not only mitochondria but also lysosomes^23,39^. Both mitochondrial defects and lysosomal function impairment can lead to neurological pathologies, suggesting functional connections between these two organelles^40,41^.

We recently demonstrated prominent mitochondrial-related defects and mitochondrial proteome perturbations in response to parkin deficiency^29^. These include disturbances in oxidative stress defense, mitochondrial respiration and morphology. Moreover, structural and functional analyses revealed an increase in mitochondrial area and dysregulation of fission-fusion dynamics^29^. In the present study, therefore, we explored the reciprocal relationship between mitochondrial and lysosomal pathways in PD by investigating the impact of parkin dysfunction on lysosome structure and function using human iPSC-derived neurons with *PARK2* mutations and isogenic healthy controls. We found striking differences in lysosomal abundance, morphology, content, and activity in the *PARK2* KO neurons. Although endo-lysosomal alterations have been documented in other parkin dysfunction models^42,43^, we believe this to be the first experimental evidence obtained from human *PARK2* KO iPSC-derived neurons.

One of the consequences of mitochondrial deficits caused by parkin dysfunction in this cellular model of PD was increased lysosomal abundance and size. This altered phenotype appeared at differentiation day 25, when the cells also exhibited aberrant mitochondria, indicating increased vulnerability of mature neurons^29^. Similar lysosomal and mitochondrial alterations to those observed in our study were detected in mitochondrial mutants exhibiting disrupted mitochondrial fission-fusion processes and cristae structure and mitochondrial impairment^25,44–46^. A recent study in mice and cellular models of mitochondrial dysfunction reported a ROS-dependent increase in numbers of lysosomes, suggesting a possible mechanism^44^. Mitochondrial activity is required to maintain proper lysosomal structure and function, as demonstrated by studies applying chemical inhibition of the electron transport chain and by in *in vitro* and *in vivo* genetic models of mitochondrial dysfunction. Specifically, disruption of mitochondrial function results in the accumulation of enlarged endo-lysosomal structures^25,44,47,48^.

Our study supports these observations as lysosomal area increased after uncoupling of mitochondrial function by CCCP treatment in both neuronal populations. CCCP-treated control neurons contained almost equal area of lysosomes per cell as *PARK2* KO untreated neurons, pointing to an association between lysosomal accumulation and mitochondrial membrane depolarization caused by CCCP. In line with this, an increase in the lysosomal level was found in HeLa cells treated with the electrogenic ionophore agents CCCP and ionomycin^49^. However, it cannot be excluded that CCCP interferes with lysosomes independently of mitochondria by impairing the vacuolar-type H^+^ -ATPase, as previously suggested, and therefore this should be further investigated^22,50^. Acute CCCP treatment was recently found to promote lysosomal biogenesis through activation of transcription factor EB (TFEB)^23,51^. TFEB regulates both mitochondrial and lysosomal biogenesis and is translocated to the nucleus upon starvation, mitophagy induction, and lysosomal functional impairment^52–54^. Whether TFEB translocation/activation is responsible for the increased lysosomal content in *PARK2* KO neurons remains to be examined.

Another contributing factor to the increased lysosomal content in *PARK2* KO neurons could be an impaired lysosomal degradation capacity and the build-up of undegraded cargo, as reported in iPSC-derived dopaminergic neurons from GBA patients^55,56^. Applying TEM we detected an increased number of large electron-lucent lysosomes in *PARK2* KO neurons. The normally dark lumen is characteristic for primary lysosomes^32^ and the observed accumulation of less electron-dense lysosomes in *PARK2* KO neurons might represent secondary lysosomes in which digestion of the contents is perturbed. Similar observations of enlarged and electron-lucent lysosomes are described in lysosomal storage disorders^57–59^. Supporting this notion, the overall lysosomal enzyme activity was significantly decreased in the *PARK2* KO neurons.

The impaired lysosomal enzyme activity we observed in *PARK2* KO neurons could be associated with autophagic disturbances as reduced autophagic flux was documented in a recent study performed on *PARK2*-PD fibroblasts and in iPSC-derived neurons from patients with GBA mutation and sporadic PD^53,60,61^. Indeed, our data showed perturbations in the total levels of LC3A and LC3B, key proteins in the autophagic process, which were significantly decreased in *PARK2* KO neurons. However, autophagic activity, as indicated by the levels of LC3A-II and LC3B-II with and without inhibition of autophagosome-lysosome fusion, was not significantly affected. For both control and *PARK2* KO neurons we found a strong tendency towards increased LC3B-II levels following bafilomycin A1 treatment, though this did not reach statistical significance. Contrarily, when analysing p62 levels under the same conditions, a significant increase was seen for control neurons with bafilomycin A1 treatment, which was not present for the *PARK2* KO neurons, pointing towards impaired autophagic degradation in the latter. Overall, this indicates that autophagic changes are present in *PARK2* KO neurons, although our results are not sufficient to determine whether autophagic flux is affected.

Lysosomal accumulation in *PARK2* KO neurons, assessed by LAMP1 staining, was increasing progressively with time. Therefore, it would be highly interesting to investigate if a similar finding is to be detected with respect to the loss of lysosomal activity and whether the autophagic perturbations are more pronounced and unambiguous at later stages.

GCase activity was significantly increased in the *PARK2* KO neurons, whereas both β-Gal activity and overall lysosomal enzymatic activity were reduced. This points to the activation of compensatory mechanisms to increase GCase activity, as the total protein level of GCase was unchanged. A direct interaction between parkin and GCase could explain such a link, as parkin can directly interact with and mediate degradation of mutant GCase^62^. This was not the case for wild type GCase, however, and further studies are needed to address the interplay between GCase activity and parkin dysfunction.

A recent study on cellular models of mitochondrial dysfunction induced by chemical stressors or deletion of mitochondrial proteins including PINK1 also documented impaired lysosomal enzyme activity. These changes were dependent on ROS levels, as antioxidant treatment could revert the observed phenotypes^25^. Given that the *PARK2* KO neurons have decreased levels of antioxidant defence enzymes^29^, a similar mechanism could be relevant in the present study. We observed significantly elevated ROS levels in our *PARK2* KO cells based on three independent differentiations, indicating that increased ROS could be a key factor here as well. However, it should be noted that when performing the NAC treatment experiment, we did not observe the baseline increase in *PARK2* KO ROS levels. We hypothesize that this discrepancy could relate to variation between batches of NSCs, causing the ROS phenotype to only be detectable at a later time point during differentiation. This is based on observations of slight variation between batches of NSCs in when certain phenotypes appear (data not shown). Despite the lacking baseline difference in ROS production in these experiments, antioxidant treatment (1 mM NAC) caused a significant reduction of ROS levels and had a beneficial effect on the mitochondrial and lysosomal area in *PARK2* KO neurons. These results support a role for enhanced ROS production as a mediator of the observed lysosomal disturbances and may potentially hint to protective properties of NAC in PD. Several studies have suggested that NAC might have a beneficial effect in neurodegenerative diseases, such as PD, through prevention of cell death caused by oxidative damage^63^ and facilitation of mitophagy^64^. However, as our observations of significantly increased ROS levels were not consistent, it cannot be excluded that the detected mitochondrial and lysosomal defects were not a direct result of ROS activity but rather a more complex mechanism of action.

## Conclusion

Our results indicate that the loss of parkin causes several lysosomal perturbations affecting their abundance, morphology, content, and activity. Taken together with our previous data, these findings indicate a causal link between two major pathological features of PD, namely mitochondrial defects and lysosomal dysregulation, supporting the idea of a direct pathogenic feedback between the two organelles. Future efforts should focus on the identifying the molecular mechanistic pathways that connect mitochondrial and lysosomal perturbations with the aim of developing therapeutic approaches for PD.

## Methods

### Ethics statement

The Research Ethics Committee of the Region of Southern Denmark approved the study prior to initiation (S-20130101). All use of human stem cells was performed in accordance with the Danish national regulations, the ethical guidelines issued by the Network of European CNS Transplantation and Restoration (NECTAR) and the International Society for Stem Cell Research (ISSCR).

### CCCP (*cyanide m-chlorophenyl hydrazine*) and NAC (*N-Acetyl-L-Cysteine*) treatments

CCCP and NAC solutions were prepared by dissolving in DMSO (all Sigma). At day 24 of differentiation cells were treated for 48 h with CCCP (10 µM), NAC (0.1 mM, 0.5 mM, 1 mM) or with equivalent dose of vehicle (DMSO).

### *In vitro* propagation and differentiation of neural stem cells (NSCs)

*PARK2* KO and healthy isogenic control NSC cell lines were provided by XCell Science Inc. (Novato, CA, USA)^30^. NSC lines were propagated according to well-established, standard protocols, using Geltrex-(Thermo Fisher) coated plates in Neurobasal Medium (Thermo Fisher) supplemented with NEAA, GlutaMax-I, B27, supplement (Thermo Fisher), penicilin-streptomycin, and bFGF. Cells were enzymatically passaged with Accutase (Thermo Fisher) when 80–90% confluent. NSCs were differentiated according to a commercially available dopaminergic differentiation kit (XCell Science Inc., Novato, CA, USA) for at least 25 days. Differentiation was divided into two parts: an induction phase, where NSCs were differentiated into dopaminergic precursors, and a maturation phase, where the dopaminergic precursor cells were differentiated into mature dopaminergic neurons. The differentiations were carried out at 37 °C in a low O_2_ environment (5% CO_2_, 92% N_2_, and 3% O_2_). The cells were seeded onto wells coated with Poly-L-Ornithine (Sigma) and Laminin (Thermo Fisher) at a density of 50,000 cells/cm^2^. Complete DOPA Induction Medium (XCell Science) supplemented with 200 ng/ml human recombinant Sonic Hedgehog (Peprotech) was changed every second day for the first nine days of differentiation. The cells were passaged at day 5 and 10 and seeded at a desired cell density. The medium was switched to Complete DOPA Maturation Medium A and B (XCell Science) at day 10 and 16, respectively.

### Quantitative real time polymerase chain reaction (qRT-PCR)

Cells were harvested using cold Trizol reagent according to the manufacturer’s instructions (Life Technologies). RNA purification was performed through columns (RNeasy Mini Kit, Qiagen) and treated with DNazeI Kit according to the manufacturer’s instructions. cDNA was synthesized using the High Capacity cDNA Archive Kit (Applied Biosystems, Thermo Fisher) according to the manufacturer’s introductions. cDNA was processed by qRT-PCR analysis using a protocol customized according to the instructions of the AB Fast-7900HT System (Applied Biosystems, Thermo Fisher). The expression level of the dopaminergic markers was analysed using the comparative method (2^-ddCt^) and quantified relative to the expression of the three housekeeping genes GAPDH, 18 S, HPRT. Relative gene expression was assessed using the following TagMan assays: *GAPDH*, Hs02758991_g1; *18 S*, Hs03003631_g1; *HPRT*, Hs02800695_m1; *EN1*, Hs00154977_m1; *NURR1*, Hs00428691_m1; *GIRK2*, Hs00158423_m1.

### Mass spectrometry (MS)-based proteomics

#### Sample preparation, labelling and enrichment of phosphorylated peptides

*PARK2* KO and control neuronal cultures were collected on ice in phosphate-buffered saline (PBS, Thermo Fisher) with protease (Complete Tablets, Roche) and phosphatase (PhosSTOP Tablets, Roche) inhibitors. The QProteome Mitochondria Isolation Kit (Qiagen) was used according to the manufacturer’s instructions and samples were denatured, reduced and enzymatically digested as previously described^29^. Peptides were desalted as previously described^29^ and labelled with Tandem Mass Tag (TMT) Sixplex Isobaric label Reagents (Thermo Fisher) according to the manufacturer’s instructions. Phospho-peptide enrichment was essentially performed as earlier described^29,65^. Mono-phosphorylated and non-modified peptides were fractionated by hydrophilic interaction liquid chromatography (HILIC) as well as high pH fractioning to increase coverage prior to liquid chromatography tandem mass spectrometry (LC–MS/MS) as described previously^29,65^.

#### LC-MS/MS

The samples were analysed by a nano-Easy LC system (Thermo Fisher Scientific) connected online to a QExactive Mass Spectrometer (Thermo Fisher Scientific). All peptide fractions were resuspended in 0.1% formic acid (FA) and loaded onto a 3 cm 100 µm inner diameter pre-column using the nano-Easy LC. The peptides were eluted directly onto the analytical column using different gradients. Depending on the samples based on the HILIC, the gradient was from 1–30% solvent B ((95% acetonitrile (ACN), 0.1% FA) for 60 or 90 min, 30–50% solvent B for 10 min, 50–100% solvent B for 5 min, 100% solvent B for 8 min. All LC–MS/MS runs were performed using an analytical column of 17 cm × 75 µm inner diameter fused silica, packed with ReproSil–Pur C18 AQ reversed-phase material (Dr. Maisch, GmbH). Mass spectrometry was performed using higher energy collision (HCD) fragmentation on a QExactive instrument. MS settings: a full MS scan in the mass range of 400–1400 Da was performed in the Orbitrap with a resolution of 120,000 full width half maximum (FWHM) and a target value of 3 × 10^6^ ions. For each full scan the 12 most abundant ions were selected for HCD fragmentation. The fragments were detected at a resolution of 60,000 FWHM for a target of 1 × 10^5^ and a maximum injection time of 60 ms using an isolation window of 1.2 m/z and a dynamic exclusion. All raw data were viewed in Thermo Xcalibur v3.0.

### MS data analysis

The raw data were processed using Proteome Discoverer (v2.1, Thermo Fisher) and searched against the Swissprot human database using an in-house Mascot server (v2.3, Matrix Science Ltd.). Databases were searched using the following parameters: precursor mass tolerance of 10 ppm, fragment mass tolerance of 0.02 Da, TMT 6-plex (Lys and N-terminal) as fixed modifications, and a maximum of two missed cleavages for trypsin. Variable modifications were NEM on Cys and N-terminal acetylation along with phosphorylation of Ser/Thr/Tyr for the phosphorylated group. Only peptides with a q-value up to 0.01 (Percolator), Mascot rank 1, and cut-off value of Mascot score > 15 were considered for further analysis. Only proteins with more than one unique peptide were considered for further analysis in the non-modified group.

### Sample preparation for transmission electron microscopy (TEM)

Cells seeded on 13 mm Thermanox plastic coverslips (Nunc) were primarily fixed in 3% glutaraldehyde (Merck) in 0.1 M sodium phosphate buffer with pH 7.2 at 4 °C for 1 h and stored in a 0.1 M Na-phosphate buffer at 4 °C until further analysis. When ready, the cells were embedded in 4% agar at 45 °C (Sigma) under the stereomicroscope and cut into 1–2 mm^3^ blocks, which were then washed with 0.1 M Na-phosphate buffer followed by post-fixation in 1% osmium tetroxide in 0.1 M Na-phosphate buffer (pH 7.2) for 1 h at room temperature (RT). Cells were washed in MilliQ water, followed by stepwise dehydration in a series of ascending ethanol concentrations ranging from 50–99% EtOH. Propylene oxide (Merck) was then used as an intermediate to allow infiltration with Epon (812 Resin, TAAB). The following day, the agar blocks were placed in flat molds in pure Epon, which was cured at 60 °C for 24 h. Approximately eight semi-thin sections (2 μm) from one block were cut on an ultramicrotome with a glass knife (Leica, Reichert Ultracut UTC). These were stained with 1% toluidine blue in 1% Borax and evaluated by light microscopy to locate areas with adequate number of cells for further processing. Ultra-thin sections (70 nm) were cut on the ultramicrotome with a diamond knife (Jumdi, 2 mm) and then collected onto TEM copper grids (Gilder) and stained with 2% uranyl acetate (Polyscience) and 1% lead citrate (Reynolds 1963). The samples were evaluated, and the images were collected using a Philips CM100 transmission electron microscope equipped with a Morada digital camera equipment and iTEM software system.

### Morphometric analysis of TEM images

Six TEM grids from each cell line were used for analysis, and ten spots were randomly chosen at low magnification. Images of each spot were captured using high magnification (19,000×). To estimate lysosomal number, all lysosomes were counted manually on each micrograph and normalized to cytoplasm area. 45 images were used for each cell line. For further measurements of area and mean grey value, individual organelles were selected and processed manually in ImageJ software using a freehand selection tool, which allows creating irregularly shaped contour. In the total 75 organelles from each cell line were analysed.

### Western blotting

Western blotting was performed using standard techniques. In brief, the cells were collected on ice in lysis buffer (PBS containing phosphatase (PhosphoSTOP Tablet, Roche) and protease (Complete Mini Tablets, Roche) inhibitors supplemented with 1% Triton X-100). Afterwards, samples were incubated for 30 min at 4 °C with shaking and centrifuged (10,000 rpm, 15 min, 4 °C), and the supernatants were collected. Samples were equalized according to the protein concentration, denatured for 10 min in NuPAGE loading buffer, separated by SDS-PAGE on NuPage 4–12% Bis-Tris or 3–8%Tris-Acetate gels (Invitrogen). The proteins separated through electrophoresis were transferred onto polyvinylidene difluoride (PVDF) membranes (Invitrogen) followed by blocking the membranes for 60 min at 4 °C with 5% non fat dry milk in a mixture of 0.05 M TBS and 0.05% Tween-20. The membranes were incubated overnight at 4 °C in solution containing relevant primary antibodies: mouse anti-TH (Chemicon/Millipore #MAB5280) 1:2000, mouse anti-MAP2a + b (Sigma #M1406) 1:500, rabbit anti-LAMP1 (Abcam #24170) 1:500, rabbit anti-LAMP2A (Abcam #18528) 1:500, rabbit anti-GBA (Abcam #154856) 1:1000, rabbit anti-LC3A (Cell Signaling #4599) 1:1000, rabbit anti-LC3B (Cell Signaling #3868) 1:1000, mouse anti-p62/SQSTM1 (Abcam #56416) 1:500, mouse anti-α-actin (Chemicon/Millipore #MAB1501) 1:6000, and mouse anti-β-actin (Abcam #49900) 1:50000. Subsequently, blots were incubated with an appropriate horseradish peroxidase (HRP)-conjugated secondary antibody (DAKO #P0260 or #P0217) with dilution 1:2000 for 1 h at RT. Protein expression was detected with ECL reagents (Thermo Fisher) using the ChemiDoc MP imaging system (BioRad), and quantified by densitometry using Image Lab software (BioRad).

### Immunofluorescence staining

Cells cultured on coverslips in 24-well plates (Costar) were fixed in 4% paraformaldehyde (PFA, Sigma) for 20 min at RT, washed twice in 0.15 M Sørensen’s buffer for 15 min and then permeabilized by washing three times in 0.1% Triton X-100 (Sigma) in 0.05 M TBS for 15 min at RT, followed by blocking in 5% goat serum (Millipore) in 0.05 M TBS for 30 min at RT. Afterwards, primary antibodies diluted in 5% goat serum were added to the cells in the following concentrations: rabbit anti-TH (Millipore #AB152) 1:600, mouse anti-MAP2a + b (Sigma #M1406) 1:2000, rabbit anti-LAMP1 (Abcam #108597) 1:1000, rabbit anti-TOM20 (Santa-Cruz #SC-11415) 1:1000. The cells were washed three times in 0.1% Triton X-100 in 0.05 M TBS for 15 min at RT and incubated with fluorophore-conjugated secondary antibodies: Alexa Fluor 488 goat anti-rabbit IgG (Invitrogen #A11008), Alexa Fluor 488 goat anti-mouse IgG (Invitrogen #A11001), Alexa Fluor 555 goat anti-rabbit IgG (Abcam #150078) or Alexa Fluor 555 goat anti-mouse IgG (Molecular probes #21422) 1:500 diluted in 5% goat serum in 0.05 M TBS for 2 h at RT. The cells were washed twice in 0.05 M TBS for 15 min at RT and then counterstained with 10 µM DAPI (Sigma) for 15 min at RT to stain all nuclei. Finally, cells were mounted onto glass slides using ProLong® Diamond mounting medium (Molecular Probes).

### Fluorescent image analysis

Fluorescence images were acquired on a FluoView FV1000MPE – Multiphoton Laser Confocal Microscope (Olympus) 20X or 60X magnification, in a blinded manner on five randomly chosen confocal fields per coverslip from independent experiments. TH + dopaminergic neurons and MAP2 + mature neurons stainings were quantified manually in ImageJ using the Cell Counter plugin. Only cells displaying an extensive immunostaining with a well-preserved cellular morphology were counted. Lysosomal and mitochondrial areas were analysed automatically in ImageJ software by converting LAMP1/TOM20 images to binary format. All stainings were normalized to total cell numbers as quantified by DAPI + nuclei analysis in CellProfiler or ImageJ software.

### ROS evaluation

ROS levels were measured using ROS-Glo™ Assay Kit (Promega) according to the manufacturer’s instructions. Briefly, cells were plated in a 96-well plate (5–10.000 cells/well). H_2_O_2_ Substrate was added to each well and incubated for 1–2 h at 37 °C. Then, ROS-Glo™ Detection Solution was added and the plate was incubated for additional 20 min at RT. The luminescence signal (RLU) was recorded using an Orion L Microplate Luminometer (Titertek Berthold). The RLU values were corrected for total cell numbers.

### Total lysosomal intracellular activity assay

Total lysosomal intracellular activity was measured using Lysosomal Intracellular Activity Assay Kit (Cell-Based) (BioVision) according to the manufacturer’s protocol. Briefly, cells were plated on 13 mm glass coverslips in a 24-well culture plate and incubated for 8 h in medium supplemented with 10% FBS at 37 °C. The media were removed and replaced with fresh aliquots supplemented with 0.5% FBS. Self-Quenched Substrate was added to each well, and the plate was incubated for 1 h at 37 °C. Ten minutes before the end of incubation, 1:1000 Hoechst solution (Sigma) was added to each well to stain nuclei. After incubation, the cells were analysed under fluorescence microscope with 488 nm excitation filter to visualize the fluorescence of released Self-Quenched Substrate, which was proportional to the total lysosomal intracellular activity.

Fluorescence images were acquired on a FluoView FV1000MPE – Multiphoton Laser Confocal Microscope (Olympus) 60X magnification. They were analysed automatically in ImageJ software by converting images to binary format. Total area of the staining was normalized to total cell numbers as quantified by Hoechst+ nuclei analysis in ImageJ software.

### β-galactosidase (β-Gal) activity assay

β-Gal activity was measured using Fluorometric β-Galactosidase Activity Assay Kit (BioVision) according to the manufacturer’s protocol. Briefly, cells were plated in a 6-well plate and homogenized with ice-cold β-Gal Assay Buffer and kept on ice for 10 min. Cell lysates were transferred to Eppendorf tubes and centrifuged at 10,000 g, 4 °C for 5 min, and then supernatants were collected. Diluted β-Gal Positive Control and supernatant were transferred in triplicates into desired wells in a 96-well black plate. The volume of samples and Positive Control wells was adjusted with β-Gal Assay Buffer. Required amount of Reaction Mix (containing β-Gal Assay Buffer and β-Gal Substrate) was prepared, and added to each well. Fluorescence signal (Ex/Em = 480/520 nm) was measured in kinetic mode, and the Fluorescein Standard Curve was read in Endpoint mode.

### β-glucocerebrosidase (GCase) activity assay

To isolate the lysosomal fraction, cell pellets, collected as earlier described, were resuspended in PBS with protease inhibitor (Complete Mini Tablets, Roche) and homogenized with a 5 ml syringe with a 21 G needle. This was followed by centrifugation (1,000 g, 10 min, 4 °C) to pellet nuclei and cell debris. The supernatant was collected and centrifuged (20,000 g, 30 min, 4 °C) to pellet the lysosomal fraction. Total cell pellets or lysosomal fraction pellets were sonicated at 10 amp for 10 sec in citrate phosphate buffer pH 5.4 consisting of 0.1 M citric acid (Sigma) and 0.2 M dibasic sodium phosphate (Sigma) with 0.25% (v/v) Triton X and 0.25% (w/v) taurocholic acid (Sigma). Samples were centrifuged at 800 g for 5 min at 4 °C, and the supernatant was collected. Then samples were diluted in citrate phosphate buffer in quadruplicate. One replicate of each sample was treated with 1 mM conduritol B epoxide (CBE, Calbiochem) for 10 min before all samples were incubated for 1 h with 2.5 mM of the fluorescent GCase substrate methylumbillifery β-D-glucopyranoside (4MUG, Sigma) at 3 °C in the dark. The reaction was quenched with 1 M glycine buffer (Sigma) pH 10.8, and the fluorescent levels were analysed on the PHERAStar FSX plate reader (BMG Labtech). Values from CBE-treated wells were subtracted as background.

### Autophagic flux

We explored whether LC3A-II, LC3B-II (markers for autophagosomal membrane) and p62 (a cargo receptor for autophagy substrates) were degraded in a lysosomal-dependent manner by using bafilomycin A1, an inhibitor of lysosomal acidification. We incubated differentiated neurons with DMSO (untreated) or medium containing bafilomycin A1 (10 nM for 6 h). Western blotting was performed on cell lysates using antibodies against LC3A, LC3B (Cell Signaling), p62 (Abcam) and α-actin (Abcam). LC3A-II, LC3B-II and p62 band intensities were quantified through densitometric analysis using ImageJ software and normalized to α-actin. Total LC3-II autophagic flux and p62 autophagic flux were calculated as the ratios LC3-II (bafilomycin A1) /LC3-II (untreated) and p62 (bafilomycin A1)/p62 (untreated), respectively.

### Statistical analysis

Data were analysed by GraphPad Prism 7.0 software using two-tailed unpaired Student’s t-test and one-way or two-way ANOVA with Tukey’s Multiple Comparison Test as appropriate. Data were considered statistically significant at p < 0.05 (*), p < 0.01 (**), and p < 0.001 (***). Data are presented as mean ± standard error of the mean (SEM).

## Supplementary information

Supplementary Information.

## Data Availability

The mass spectrometry proteomics data have been deposited to the ProteomeXchange Consortium via the PRIDE partner repository with the dataset identifier PXD008894. The remaining datasets used and/or analysed during the current study are available from the corresponding author on request.
