# Supplementary material for: Lysosomal perturbations in human dopaminergic neurons derived from induced pluripotent stem cells with PARK2 mutation
Source: Sci Rep. 2020 Jun 24;10:10278. doi: 10.1038/s41598-020-67091-6 (PMC7314796; doi:10.1038/s41598-020-67091-6)
Supplement: Supplementary file 1 — Supplementary Information. [file 41598_2020_67091_MOESM1_ESM.docx]

**Revised Supplementary Information**

**Lysosomal perturbations in human dopaminergic neurons derived from induced pluripotent stem cells with *PARK2* mutation**

Justyna Okarmus^1,†^, Helle Bogetofte^1,†^, Sissel Ida Schmidt^1^, Matias Ryding^1^, Silvia García-López ^2^, Brent James Ryan^3^, Alberto Martínez-Serrano^2^, Poul Hyttel^4^, Morten Meyer^1,5,*^

*^1^Department of Neurobiology Research, Institute of Molecular Medicine, University of Southern Denmark, J.B. Winsløws Vej 21, 5000 Odense C, Denmark; ^2^Department of Molecular Biology and Center of Molecular Biology Severo Ochoa, Autonomous University of Madrid-C.S.I.C Campus Cantoblanco, Madrid, Spain; ^3^Oxford Parkinson’s Disease Centre, Department of Physiology, Anatomy and Genetics, University of Oxford, Oxford, United Kingdom; ^4^Department of Veterinary and Animal Sciences, Faculty of Health and Medical Sciences, University of Copenhagen, Grønnegaardsvej 7, 1870 Frederiksberg C, Denmark; ^5^BRIDGE – Brain Research Inter-Disciplinary Guided Excellence, Department of Clinical Research, University of Southern Denmark, J.B. Winsløws Vej 19, 5000 Odense C, Denmark*

^†^ *Authors contributed equally to this work*

* Corresponding author:

Morten Meyer, Ph.D.

Department of Neurobiology Research

Institute of Molecular Medicine

University of Southern Denmark

J.B. Winsløws Vej 21, st

5000 Odense C, Denmark

Telephone: +45 65503802

E-mail: [mmeyer@health.sdu.dk](mailto:mmeyer@health.sdu.dk)


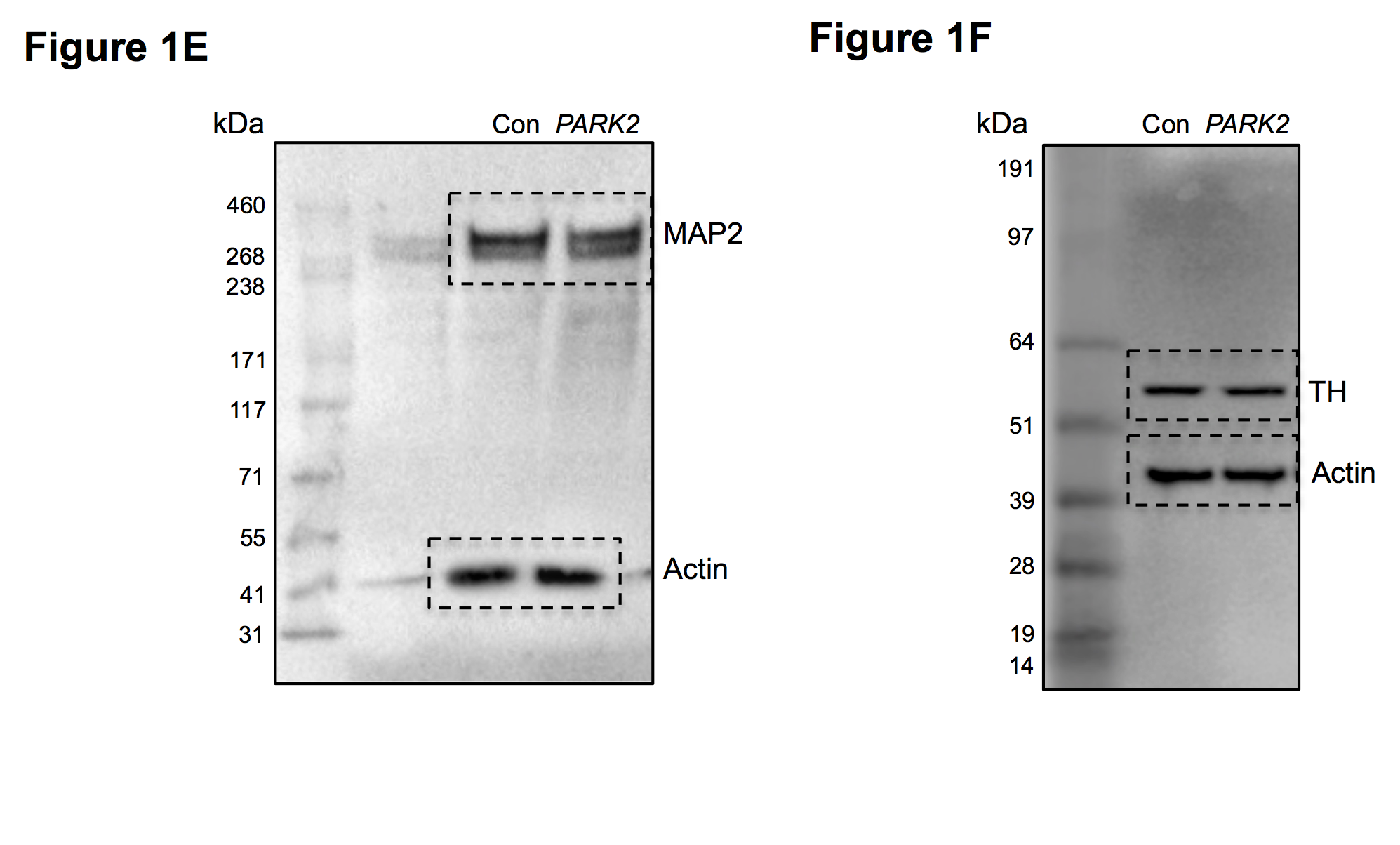


Supplementary Figure S1: Full-size Western blots for MAP2 and TH.

Full-length blots referring to main Fig. 1E and F. Dashed black boxes indicate the regions selected for presentation in Fig. 1E and F.


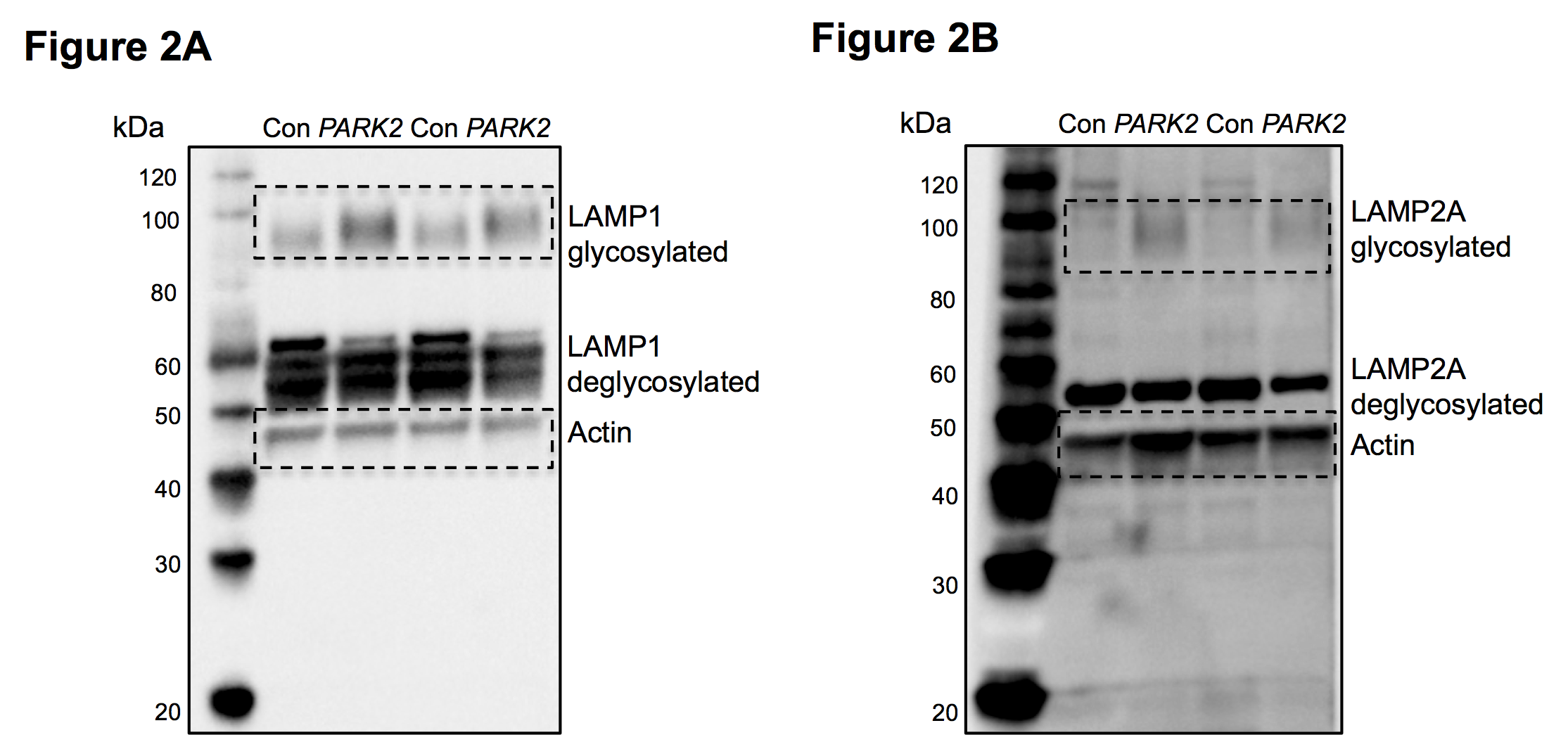


Supplementary Figure S2: Full-size Western blots for LAMP1 and LAMP2A.

Full-length blots referring to main Fig. 2A and B. Dashed black boxes indicate the regions selected for presentation in Fig. 2A and B.


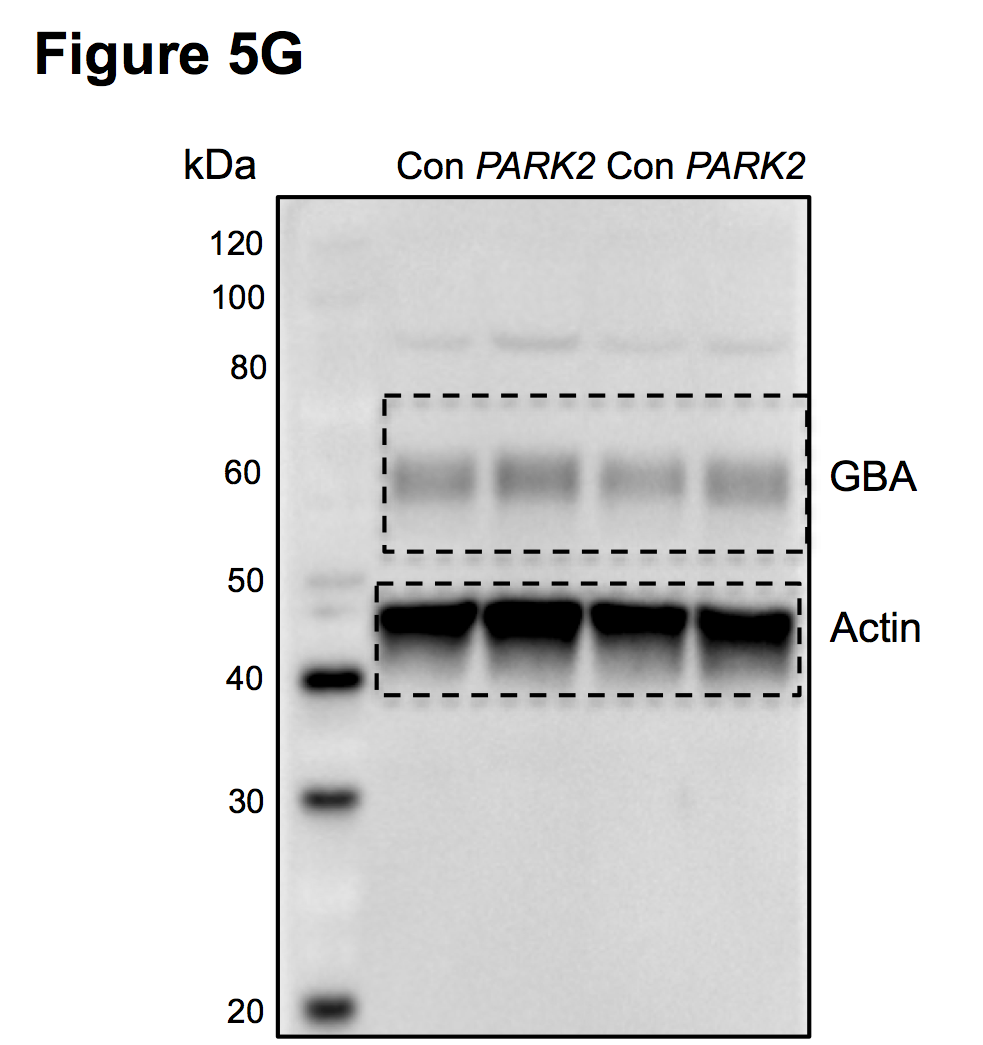


Supplementary Figure S3: Full-size Western blots for GBA.

Full-length blot referring to main Fig. 5G. Dashed black boxes indicate the regions selected for presentation in Fig. 5G.


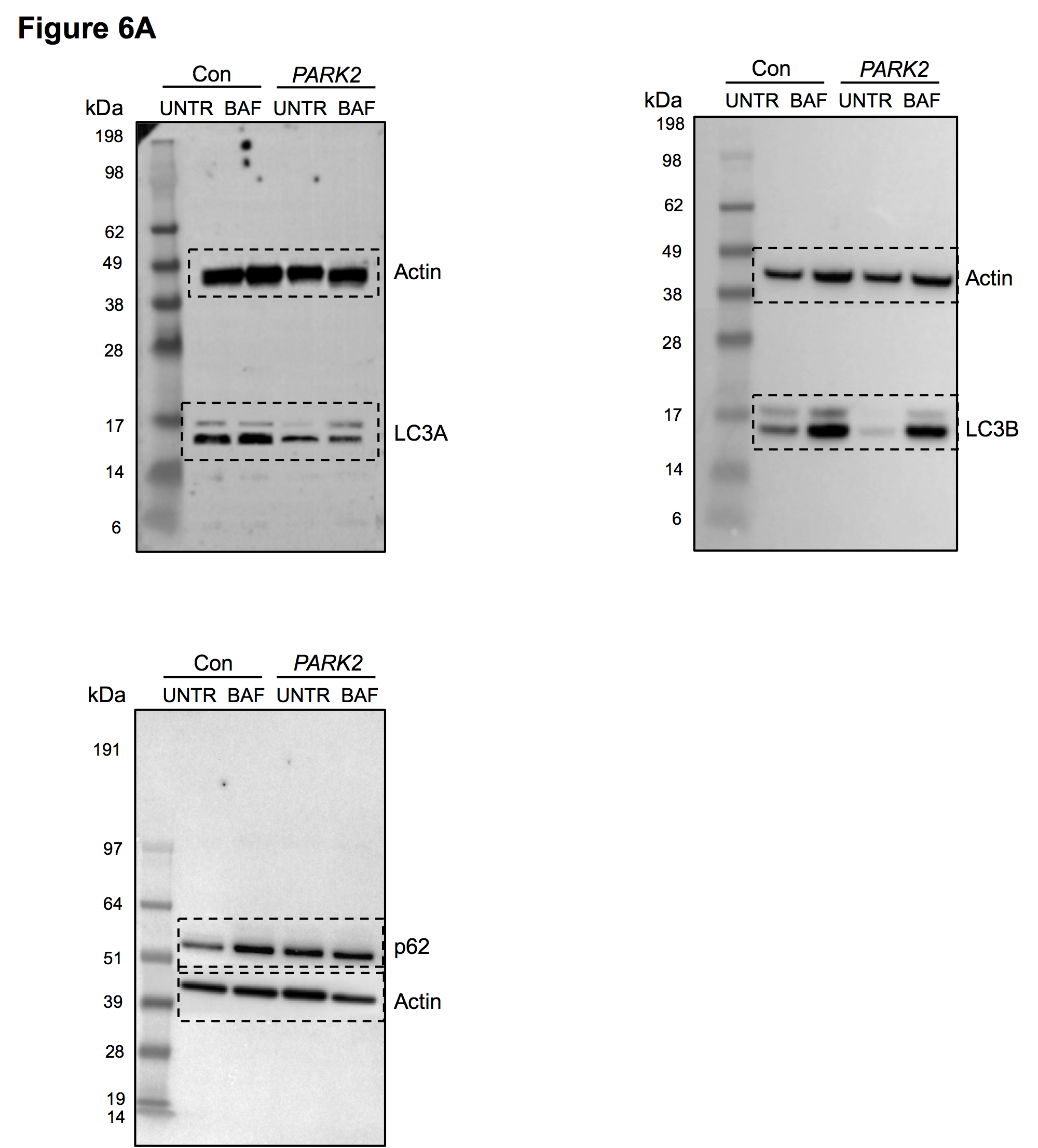


Supplementary Figure S4: Full-size Western blots for LC3A, LC3B and p62.

Full-length blots referring to main Fig. 6A. Dashed black boxes indicate the regions selected for presentation in Fig. 6A.

Supplementary Figure S5: Effect of the mitochondrial uncoupler CCCP (10 μM) on the total number of cells after 24, 48, 72, and 96 hrs exposure.

The figure refers to data presented in Figs. 4D, E.


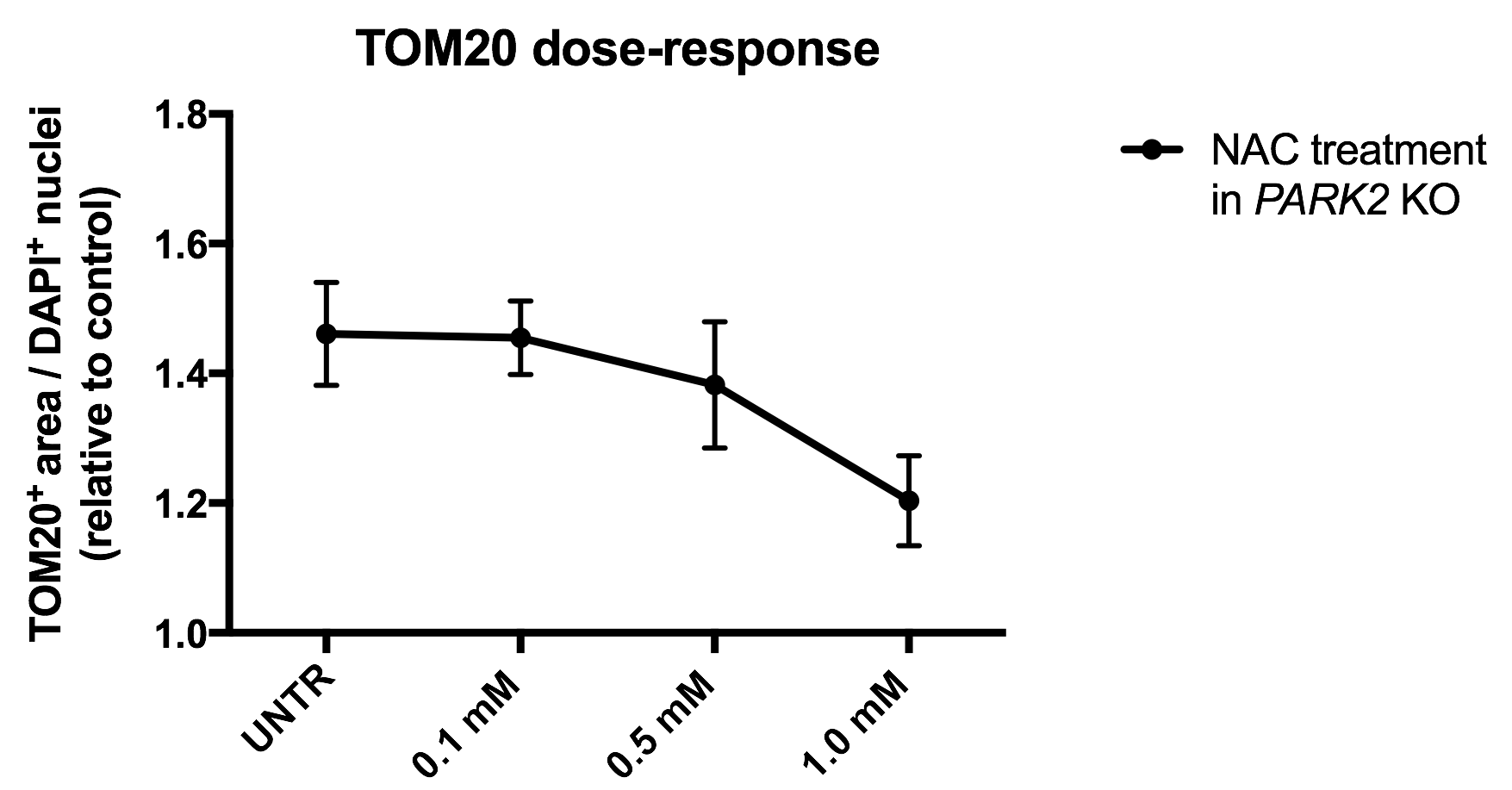


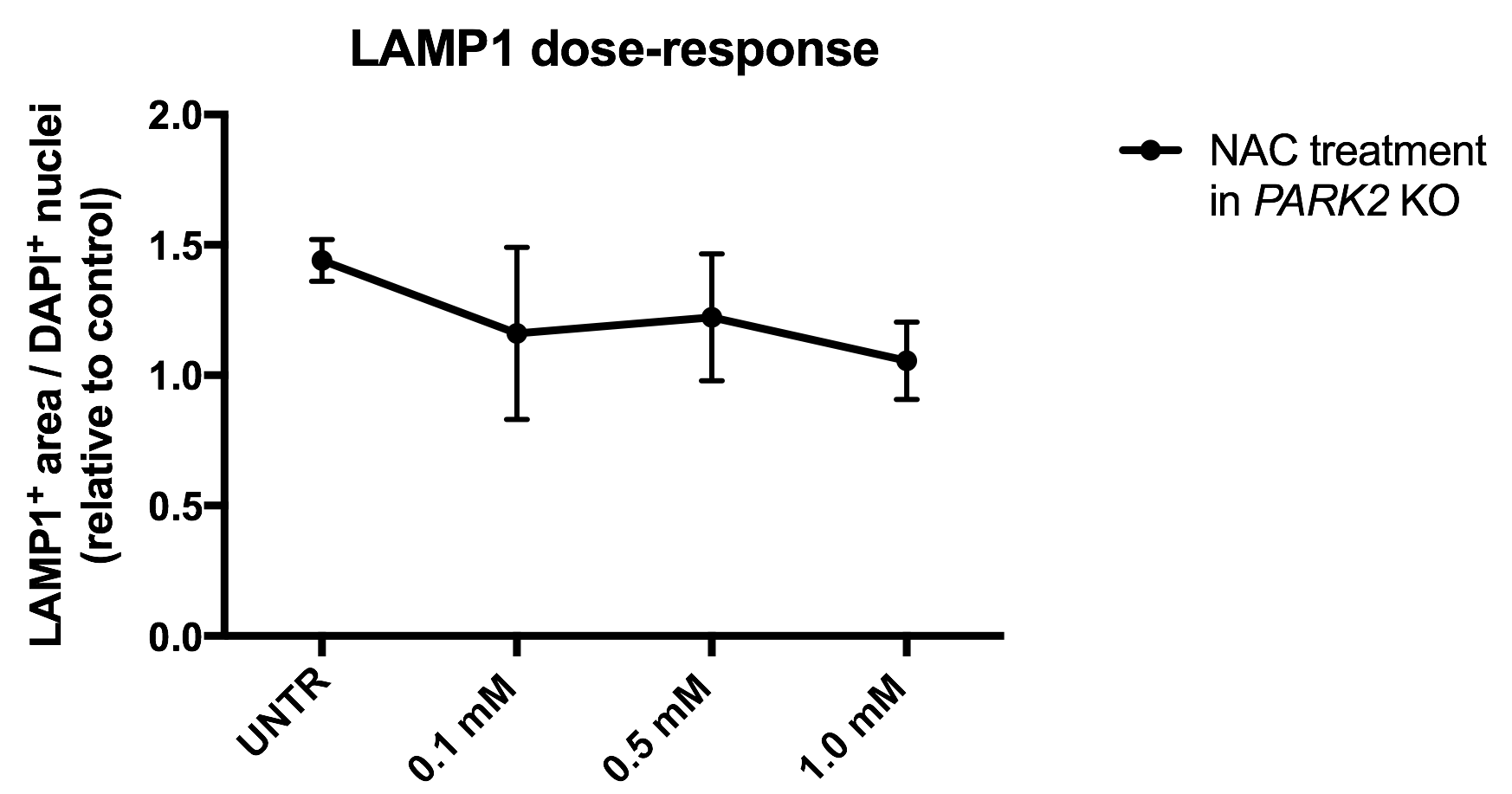


Supplementary Figure S6: Dose-response curves presenting effects of NAC (0.1 mM, 0.5 mM, 1mM) treatment on TOM20+ mitochondrial and LAMP1+ lysosomal areas after 48h exposure. The figure refers to data presented in Figs. 7B-F.
